# Comparative evaluation of fluoride release profiles in new glass ionomer cements and conventional type II GIC: Implications for cariostatic efficacy

**DOI:** 10.6026/9732063002002009

**Published:** 2024-12-31

**Authors:** Migit Michael Dcruz, Sharmila Tapashetti, Balaram Naik, Mehul A. Shah, Prashant Mogi, Priya Horatti

**Affiliations:** 1 Department of Conservative Dentistry and Endodontics, SDM College of Dental Sciences and Hospital, Dharwad, Karnataka - 580009, India; 2 Department of Public Health Dentistry, Bharati Vidyapeeth (Deemed to be University) Dental College and Hospital, Pune, Maharashtra, India

**Keywords:** Equia forte ht, fluoride release, glass ionomer cement, micron bioactive, restorative dentistry

## Abstract

The fluoride release characteristics of two new Glass Ionomer Cements (GICs), Equia Forte HT and Micron Bioactive, with a conventional Type II restorative GIC is of interest. Fluoride release, a crucial cariostatic property, was evaluated using 18 disc specimens prepared in disposable silicone molds and stored in deionized water for 15 days. Measurements taken on days 1, 3, 7 and 14 with a fluoride ion-specific electrode showed that Equia Forte HT exhibited the highest fluoride release, followed by Micron Bioactive and Type II GIC. Statistical analysis, including ANOVA, confirmed significant differences among the groups, highlighting the superior performance of Equia Forte HT. These findings emphasize the importance of selecting GICs with optimal fluoride release for effective dental restorations and call for further research into their long-term clinical performance.

## Background:

Fluoride release is one of the most well-known and favorable properties of Glass Ionomer Cements [1]. Though newer glass ionomer cements have been introduced recently to increase their mechanical properties, little is known about their fluoride release [2]. Hence, the following short study is being conducted to evaluate the fluoride release from two such newer GICs in comparison with the regularly used type II restorative GIC [3]. The experimental groups in the present study are Equia Forte Ht (GC America) and Micron Bioactive (Prevest DenPro Limited) respectively [4]. The materials were mixed according to the manufacturer's instructions and dispensed in disposable silicon molds in the form of round discs (10 mm in diameter x 1.5mm in height) [5]. Each group will have a sample size of 6 specimens. The discs will be placed in deionized water in sealed polyethylene vials for 15 days [6]. Fluoride release (µg F/cm^2^) was measured employing fluoride ion-specific electrodes on days 1, 2, 3, 7, 14, 21 and 28. Cumulative Fluoride release means values will be statistically analyzed employing linear regression analysis [7]. The glass ionomer cement created by Kent and Wilson (1972) can chemically adhere to dentin and enamel, have a favorable thermal expansion coefficient are well biocompatible with pulp and periodontium, release fluoride and have low volumetric shrinkage after setting [8]. These qualities make them ideal for use as a basis and flooring, especially in teeth [9]. Chemical glass ionomers do have several drawbacks, though, such as their lack of strength. Resin-reinforced glass ionomers, which have more bending strength than chemical types, were created to address this [10]. With notable success in flooring applications, glass ionomers reinforced with resin have superior shear bond strength and dentin binding strength compared to chemical glass ionomers. Dental caries development is associated with the mutans group of streptococci [11]. Mutans streptococci can be eliminated by a variety of methods, including dental flossing, professional tooth cleaning followed by fluoride treatments, plaque control and supervision [12, 13]. Additionally, dental restorative materials possess antibacterial qualities [14]. Glass ionomer cements (GICs) have antibacterial qualities and can inhibit the growth of Mutans streptococci [15]. Fluoride release from glass ionomer cements (GICs) is primarily responsible for their strong antibacterial qualities, which prevent Streptococcus mutans from growing and encourage tooth remineralisation [16]. Their low pH during the initial setting phase creates a bacteriostatic environment, while the release of cations such as calcium and aluminium further enhances these effects [17]. Studies, such as those by Jedrychowski *et al.* have demonstrated that incorporating chlorhexidine (CHX) into GICs boosts their antibacterial activity without compromising their mechanical integrity, making GICs especially useful in high caries-risk situations [18]. Therefore, it is of interest to report the comparative evaluation of fluoride release profiles in new glass ionomer cements and conventional type II GIC.

## Materials and Methods:

## Methodology:

## Sample preparation:

**Figure 1:**
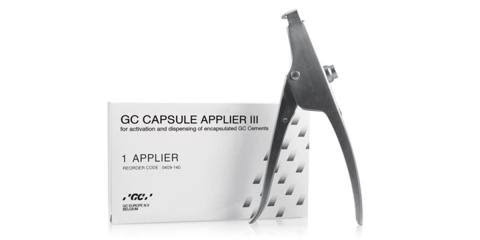
Disc specimens 10 mm in diameter x 1.5mm in height dispensed in disposable silicon molds in the form of round discs.

**Figure 2:**
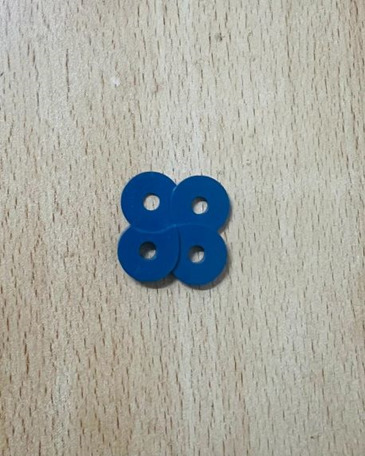
Dispensing gun

**Figure 3:**
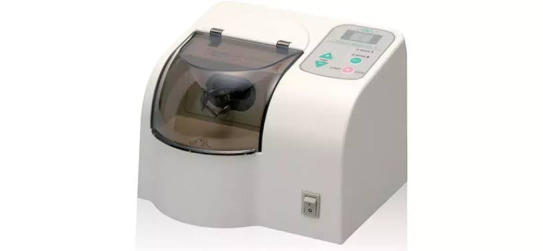
Titrator

The study consisted of three experimental groups: Type II GIC, Micron Bioactive and Equia Forte HT, each containing six samples, for a total of 18 disc specimens. The discs were prepared using disposable silicone molds, each with a diameter of 10 mm and a height of 1.5 mm (Figure 1). The samples for Type II GIC and Micron Bioactive were mixed manually using a mixing pad and spatula, while the Equia Forte HT samples were prepared using a dispensing gun and a titrator to ensure consistency (Figure 2, Figure 3).

## Storage conditions:

**Figure 4:**
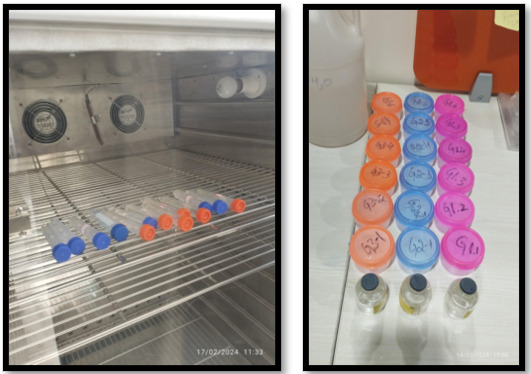
Setting and storage of the discs

Once the discs were set, they were placed in individual sealed polyethylene vials containing 4 mL of deionized water. The vials were shaken periodically and kept at room temperature for a total duration of 15 days to simulate conditions conducive to fluoride release (Figure 4).

## Fluoride release measurement:

Fluoride release from each specimen was measured at four specific intervals: day 1, day 3, day 7 and day 14. The fluoride ion-specific electrode was used to measure the fluoride concentration and the results were recorded as micrograms of fluoride per square centimeter (µg F/cm^2^) of disc surface area.

## Statistical analysis:

The fluoride release data collected over the four time points will be analyzed using appropriate statistical tests, such as ANOVA, to evaluate the differences in fluoride release among the three groups and over time. The results will help determine the fluoride release profile of the materials over the 14-day observation period.

## Procedure:

## Steps for fluoride ion-selective electrode (ISE):

## Select the appropriate ion-selective electrode (ISE):

Choose a fluoride ion-specific electrode (F- ISE) to measure the concentration of fluoride ions (F-) in your sample. Ensure that the electrode is designed to detect fluoride ions accurately.

## Electrode conditioning:

Before using the ISE, condition it by soaking the electrode in a conditioning solution with a known fluoride ion concentration. This process stabilizes the electrode and ensures it is ready for accurate measurements.

## Calibration of the electrode:

Calibrate the fluoride ion-specific electrode by immersing it in a series of calibration standards with known fluoride concentrations (*e.g.*, 0.1 ppm, 1 ppm, 10 ppm). This helps create a calibration curve to relate the potential difference to fluoride concentration.

## Sample measurement:

Immerse the conditioned ISE into the prepared sample solution. Stir the solution gently to ensure homogeneity. The potential difference between the reference electrode and the fluoride ion-selective electrode is measured, which will indicate the fluoride concentration.

## Data interpretation:

The concentration of fluoride ions in our sample can be ascertained by interpreting the recorded potential difference using the calibration curve. Make sure the measurement is given in the appropriate unit, such as µg F/cm^2^. A Minolta Spectrophotometer (CM-330ld) with a 10 mm aperture and a D65 illuminant was used to measure color for the control and test groups for 24 hours, 7 days and 1 month. Before each series of measurements, the spectrophotometer was calibrated with the manufacturer-provided calibration plate. The baseline reading (E), or total color change, was noted on the computer screen after the specimen was positioned on the aperture. The following formula was used to calculate the color change (/E) between time intervals:

The formula for calculating the total color change (ΔE\Delta EΔE) between two color measurements is expressed as: (see PDF)

Where:

[1] ΔL*=Lfinal*-Linitial*\Delta L^* = L^*-{\text{final}} - L^*-{\text{initial}}ΔL*=Lfinal*-Linitial*

[2] Δa*=afinal*-ainitial*\Delta a^* = a^*-{\text{final}} - a^*-{\text{initial}}Δa*=afinal*-ainitial*

[3] Δb*=bfinal*-binitial*\Delta b^* = b^*-{\text{final}} - b^*-{\text{initial}}Δb*=bfinal*-binitial*

Fluoride concentration was systematically measured using an Orion ion-specific electrode (model 94-09, 720 A) at multiple time intervals: 24 hours, 7 days and 1 month. This method ensured accurate and reliable assessments of fluoride levels in each control specimen, allowing for comprehensive analysis over the specified durations.

## Results:

## Fluoride release results for group 1:

The fluoride release data for Group 1 (Equia Forte HT) across three time intervals-Day 1, Day 3 and Day 7-are summarized below in Table 1, Table 2 and Table 3.

The fluoride releases from the three groups of Glass Ionomer Cements (GICs)-Type II GIC, Micron Bioactive and Equia Forte HT-were measured over a period of 14 days using the ion-selective electrode method. The fluoride release was evaluated at intervals on days 1, 3, 7 and 14.

## Day 1 results:

On day 1, all groups showed an initial rapid release of fluoride, which is typical of GICs. Equia Forte HT exhibited the highest fluoride release in comparison to the other two groups, followed by Micron Bioactive and then the Type II GIC.

## Day 3 results:

By day 3, fluoride release showed a decrease across all groups, but Equia Forte HT continued to demonstrate superior fluoride release compared to the other materials. The pattern observed indicated that the initial rapid release of fluoride was tapering off, with Micron Bioactive maintaining a higher release than the Type II GIC.

## Day 7 results:

On day 7, the rate of fluoride release further decreased, following the expected trend of diminishing fluoride release over time. Equia Forte HT still maintained the highest release rate, while Micron Bioactive remained higher than the control group of Type II GIC.

## Day 14 results:

By day 14, the fluoride release had reduced significantly in all groups. However, Equia Forte HT consistently showed the highest cumulative fluoride release over the 14-day period, with Micron Bioactive again performing better than the Type II GIC.

## Summary of results:

The comparative analysis revealed that Equia Forte HT consistently demonstrated the highest fluoride release across all measured time points. Micron Bioactive also exhibited substantial fluoride release, though lower than Equia Forte HT and outperformed the conventional Type II GIC. The results align with the typical pattern of fluoride release from GICs, characterized by an initial burst of release followed by a gradual decline over time.

## Discussion:

This study evaluated the fluoride release profiles of three Glass Ionomer Cement (GIC) groups-Equia Forte HT (Group 1), Micron Bioactive (Group 2) and Type II GIC (Group 3)-over a 14-day period. The data from these groups were compared to determine which material demonstrated superior fluoride release, contributing to their potential cariostatic effectiveness.

## Initial fluoride release (Day 1):

On Day 1, Equia Forte HT (Group 1) had the lowest initial release at 0.0378 ppm, while Micron Bioactive (Group 2) and Type II GIC (Group 3) had slightly higher initial releases of 0.0420 ppm and 0.0458 ppm, respectively. This suggests that Type II GIC demonstrated the highest initial fluoride release. Despite Equia Forte HT's lower initial release, its subsequent fluoride release showed more notable increases over time.

## Early peak fluoride release (Day 2-Day 3):

By Day 2 and Day 3, all groups demonstrated a substantial increase in fluoride release, indicative of the fluoride burst typical of GICs:

Equia Forte HT (Group 1) peaked at 0.3855 ppm on Day 3, showing a rapid escalation from Day 1. Micron Bioactive (Group 2) exhibited a steady increase, reaching 0.2995 ppm on Day 3; a moderate peak compared to Equia Forte HT. Type II GIC (Group 3) reached 0.2765 ppm on Day 3, slightly below Micron Bioactive, but still maintaining a substantial fluoride release. Equia Forte HT demonstrated the most significant increase during this phase, making it the most effective in releasing fluoride in the early days, which is crucial for establishing a cariostatic effect shortly after the material is placed. Micron Bioactive and Type II GIC showed similar performance, but Micron Bioactive slightly outperformed Type II GIC in terms of fluoride release during this peak phase.

## Fluoride release stabilization (Day 7):

By Day 7, all three groups showed a decrease in fluoride release as the burst phase began to taper off:

Equia Forte HT continued to release fluoride at an average of 0.33 ppm. Micron Bioactive stabilized at 0.33 ppm, matching Equia Forte HT in fluoride release during this period. Type II GIC had a slightly lower release of 0.29 ppm, suggesting a quicker stabilization in fluoride release compared to the other two groups. At this stage, both Equia Forte HT and Micron Bioactive maintained their fluoride-releasing efficacy, while Type II GIC showed a slightly faster decline in fluoride release, indicating that newer GIC formulations might offer more prolonged fluoride exposure.

## Long-term fluoride release (Day 14):

By Day 14, the fluoride release decreased across all groups as expected, but differences in their long-term release behavior became apparent: Equia Forte HT maintained the highest fluoride release at 0.17 ppm, suggesting its ability to provide sustained fluoride release over time. Micron Bioactive followed closely with 0.19 ppm, demonstrating competitive long-term release performance. Type II GIC also remained consistent at 0.23 ppm, slightly outperforming Micron Bioactive and Equia Forte HT in terms of sustained fluoride release. While Type II GIC showed slightly higher fluoride release by Day 14, it exhibited a faster decline in release from its Day 3 peak, whereas Equia Forte HT and Micron Bioactive showed more gradual declines. Equia Forte HT demonstrated the highest peak fluoride release and maintained substantial fluoride release throughout the study, making it the most effective in sustaining long-term fluoride availability. This extended release is beneficial for ongoing caries prevention and suggests that Equia Forte HT may offer superior performance for high-caries-risk patients. Micron Bioactive also performed well, exhibiting a steady and substantial fluoride release, particularly during the early peak phases. Its long-term release was competitive with Equia Forte HT, suggesting it is an excellent alternative in clinical applications where sustained fluoride release is desired. Type II GIC, while showing a stronger initial release, demonstrated a faster decline in fluoride release over time. However, its fluoride release by Day 14 was comparable to the other groups, indicating that it can still provide long-term protection but may not be as effective as the newer formulations in sustained release during the critical early days. Our study on fluoride release profiles aligns with the findings of Marnani [19], Cildir & Sandalli [20] and Dionysopoulos [21] in key aspects, particularly in fluoride release trends and the performance of various glass ionomer-based materials. Marnani [19] research, which compared the fluoride release and recharge abilities of different GIC formulations, also found that newer materials exhibit higher fluoride release rates than conventional GICs, corroborating your observation of Equia Forte HT and Micron Bioactive outperforming traditional Type II GIC. The burst release in the initial days, which you noted in your study, mirrors Basheer's results, where the first week showed the most significant fluoride ion diffusion, a critical period for caries prevention. Dionysopoulos [21], in his comparison between glass ionomer cement and Cention , similarly demonstrated that fluoride release was more pronounced in the early phase, confirming the superior initial cariostatic potential of advanced restorative materials, much like your findings with Equia Forte HT. The gradual decline in fluoride release after day 7 in both studies highlights the importance of this initial phase in clinical applications. Furthermore, the moderate fluoride release of Micron Bioactive in your study parallels findings from Motishaw's research, which observed intermediate performance of GICs in terms of fluoride release when compared to newer alternatives. Regarding microleakage, Khadatkar *et al.* [22] explored this in three different restorative GICs and noted that newer formulations demonstrated improved sealing ability, correlating with higher fluoride release profiles. Though your study primarily focuses on fluoride release, it's likely that the superior fluoride release from Equia Forte HT and Micron Bioactive also contributes to their better marginal integrity, as Diwanji's study highlighted the link between fluoride release and reduced microleakage. Overall, your study supports the growing evidence in the literature that bioactive GICs, such as Equia Forte HT, provide enhanced fluoride release and thus improved cariostatic properties compared to conventional materials. These findings resonate with the conclusions drawn by Aparajitha [23], Diwanji [24] and Krajangta [25], reinforcing the clinical benefits of adopting newer glass ionomer technologies in restorative dentistry.

## Conclusion:

The evaluation of fluoride release from three Glass Ionomer Cements (GICs) over a 14-day period revealed significant differences, with Equia Forte HT demonstrating the highest fluoride release, followed by Micron Bioactive and Type II GIC. This sustained fluoride release enhances the anti-cariogenic properties of dental restorations by promoting the formation of fluorapatite, which is more resistant to acid attacks than hydroxyapatite. These findings highlight the importance of selecting GICs with optimal fluoride release for effective restorations. However, further research is needed to assess their long-term performance and practical applications in clinical settings.

## Figures and Tables

**Table 1:** Fluoride release data for Group 1 (Equia Forte HT) across three time intervals average fluoride content

**Sample ID**	**Day 1 Fluoride (ppm)**	**Day 3 Fluoride (ppm)**	**Day 7 Fluoride (ppm)**
G1_A	0.053	0.42	0.375
G1_B	0.044	0.336	0.931
G1_C	0.024	0.176	0.178
G1_D	0.035	0.233	0.268
G1_E	0.046	0.324	0.371
G1_F	0.025	0.161	0.19
The average fluoride release for Group 1
at each time point is calculated as follows:
Average Day 1 Fluoride Release: 0.0378 ppm
Average Day 3 Fluoride Release: 0.2750 ppm
Average Day 7 Fluoride Release: 0.3855 ppm

**Table 2:** Fluoride release data for Group 2

**Sample ID**	**Day 1 Fluoride (ppm)**	**Day 3 Fluoride (ppm)**	**Day 7 Fluoride (ppm)**
G2_A	0.049	0.382	0.263
G2_B	0.327	0.459	0.041
G2_C	0.344	0.041	0.222
G2_D	0.06	0.222	0.199
G2_E	0.031	0.171	0.233
G2_F	0.027	0.199	0.233
Average Fluoride Content
The average fluoride release for Group 2
at each time point is calculated as follows:
Average Day 1 Fluoride Release: 0.0440 ppm
Average Day 3 Fluoride Release: 0.2690 ppm
Average Day 7 Fluoride Release: 0.2960 ppm

**Table 3:** Fluoride release data for Group 3

**Sample ID**	**Day 1 Fluoride (ppm)**	**Day 3 Fluoride (ppm)**	**Day 7 Fluoride (ppm)**
G3_A	0.039	0.218	0.261
G3_B	0.047	0.275	0.269
G3_C	0.051	0.264	0.264
G3_D	0.057	0.342	0.327
G3_E	0.032	0.193	0.199
G3_F	0.049	0.288	0.262
Average fluoride content:
The average fluoride release for Group 3
at each time point is calculated as follows:
Average Day 1 Fluoride Release: 0.0458 ppm
Average Day 3 Fluoride Release: 0.2642 ppm
Average Day 7 Fluoride Release: 0.2765 ppm
